# Antwort auf den Kommentar von Hauch et al. zum Beitrag: „PraeKids: Diagnoseprävalenz lebensbedrohlicher und lebensverkürzender Erkrankungen bei Kindern und Jugendlichen in Deutschland“: Bundesgesundheitsbl – Gesundheitsforsch – Gesundheitsschutz (Heft 7/2023)

**DOI:** 10.1007/s00103-023-03796-z

**Published:** 2023-11-16

**Authors:** Nadja Melina Burgio, Sven Jennessen

**Affiliations:** Abteilung Pädagogik bei Beeinträchtigungen der körperlich-motorischen Entwicklung, Institut für Rehabilitationswissenschaften, Kultur‑, Sozial- und Bildungswissenschaftliche Fakultät, Humboldt-Universität zu Berlin, Unter den Linden 6, 10099 Berlin, Deutschland

Zunächst möchten sich die Autor:innen bei den Kolleg:innen für den Kommentar und die damit verbundene ausführliche Auseinandersetzung mit der Studie PraeKids und dem auf dieser Grundlage entstandenen, die zentralen Erkenntnisse beinhaltenden Fachartikel bedanken. Die Autor:innen sind selbstverständlich an einem fachlichen Diskurs interessiert und nehmen gerne zu den genannten Anmerkungen zur Studie Stellung.

## Zu: Allgemeines

Zunächst erscheint es wichtig, an dieser Stelle erneut auf die Zielsetzung der Untersuchung zu verweisen. Im Rahmen der PraeKids-Studie wurde die Diagnoseprävalenz von Kindern und Jugendlichen im Alter von 0–19 Jahren mit lebensbedrohlichen und lebensverkürzenden Erkrankungen in Deutschland erhoben. Zielgruppe und fokussierte Altersspanne wurden im zugehörigen Artikel dezidiert erläutert und begründet. Es handelt sich demnach nicht um eine Erhebung der Prävalenz von Erkrankungen bei Kindern und Jugendlichen, die zum Erhebungszeitpunkt einen palliativen Versorgungs- oder hospizlichen Begleitungsbedarf haben.

So wird in dem kommentierten Beitrag erläutert, dass in den TfSL^1^-Gruppen 1 und 4 Diagnosen subsumiert sind, die potenziell lebensbedrohlich sind, aber nicht zwangsläufig zu einer Lebensverkürzung führen müssen. Dieser relevanten Differenzierung wird explizit durch den Verweis Rechnung getragen, dass die transparent erfassten (und nicht wie im Kommentar beschrieben „geschätzten“) Daten keine Aussagen zu quantitativen Versorgungsbedarfen der Zielgruppe ermöglichen. Der ebenfalls im Kommentar formulierte Hinweis, dass damit „Bedarfe für diese vulnerable Gruppe unserer Gesellschaft formuliert werden können“, entspricht somit nicht dem Erkenntnisinteresse der Studie. Zudem wäre es aus wissenschaftlicher Sicht fragwürdig, die unter konsequenter Beachtung wissenschaftlicher Gütekriterien erfassten Daten aus Gründen ihrer möglichen Fehlinterpretation nicht zu veröffentlichen und der Fachcommunity somit als Basis für weitere relevante Studien vorzuenthalten.

Bei der Angabe von 50.000 Betroffenen beziehen wir uns auf die in der hospizlichen und palliativen Versorgungslandschaft sowie der Literatur aktuell kommunizierte Zahl, der lediglich ein unbereinigter, statistischen Transfer englischer Daten auf die Anzahl von Kindern und Jugendlichen bis 19 Jahren in Deutschland zugrunde liegt [1–3]. Diese Zahl und auch die ursprünglich zugrunde liegende Studie von Fraser et al. [4] waren im Rahmen von PraeKids Ausgangspunkt für die Konzeption des Studiendesigns und der Erhebung der Diagnoseprävalenz. Im Artikel wurde darauf hingewiesen, dass die Zahl 50.000 aufgrund der aktuellen Ergebnisse von PraeKids und der im Bericht zitierten internationalen Studien [5, 6], die einen epidemiologischen Vergleich zwischen England und Deutschland eher kritisch betrachten lassen, überdacht werden sollte. Dementsprechend erscheint eine erneute Hochrechnung englischer Prävalenzen auf die im Kommentar genannte Zahl von 100.000 nicht sinnvoll, da sämtliche länderspezifische Faktoren (Sterblichkeitsrate, Armut, Anteil an Kindern aus Einwandererfamilien etc.) unberücksichtigt bleiben. Neben der Tatsache, dass bei einem solchen Transfer die bei PraeKids einbezogenen, in den letzten Jahren ergänzten Codes unberücksichtigt wären, würden durch dieses Vorgehen die ICD-10-Codes (Internationale statistische Klassifikation der Krankheiten und verwandter Gesundheitsprobleme) aus der Fraser-Liste [4] in die Berechnung einfließen, die durch Palliativmediziner:innen in der Gruppendiskussion als weder lebensbedrohlich noch lebensverkürzend eingestuft wurde (s. Fachartikel und Tab. 1), z. B. H11.1 *Konjunktivadegeneration und -einlagerungen* oder E80.3 *Erworbene Aphasie mit Epilepsie (Landau-Kleffner-Syndrom*).

Wir beziehen uns in unserer Studie auf die Untersuchungen von Fraser et al. aus den Jahren 2012 und 2020 [4, 7], da die in der deutschen Versorgungslandschaft kommunizierte Zahl von 50.000 Kindern und Jugendlichen auf die in diesen Studien verwendete Altersrange (0–19 Jahre) rekurriert. Auch in unserem Verweis der unterschiedlichen Prävalenzanstiege in England und Deutschland nutzen wir als Referenz die Veröffentlichung von Fraser et al. [7] die 0‑ bis 19-Jährige einbezogen haben. Die mit dem Hinweis auf die Studie von Fraser et al. verbundene Kritik an der in PraeKids fokussierten Altersrange ist somit nicht nachvollziehbar.

## Zu: Grundlegende Unterschiede in der Methodik

Es wird bemängelt, dass die verwendeten stationären Daten des Instituts für angewandte Gesundheitsforschung (InGef) nicht ausgewiesen wurden. Mit Blick auf die Erhebung der Gesamtprävalenz haben die Autor:innen für diese Studie bei den Fallerhebungen beide Versorgungssettings pro Kind/Jugendliche:r einbezogen, sofern eine Versorgung sowohl im ambulanten als auch stationären Bereich erfolgte. Welches Einschlussverfahren hinsichtlich der Datensettings für GKV-SV (Spitzenverband Bund der Krankenkassen) und InGef genutzt wurde, wird anhand der Beschreibungen der Methodik und der Diskussion der Ergebnisse transparent kommuniziert. Zudem ist anzumerken, dass in PraeKids ausschließlich die Prävalenz der ausgewählten Diagnosen erhoben wurde, weshalb die Schwere der Erkrankungen, die im Kommentar zu Recht vermehrt im stationären Bereich gesehen wird, für den Versorgungsbedarf relevant ist, nicht jedoch für das beschriebene Studienziel.

Die möglichen Verzerrungen der GKV-Stichprobe und die Kritik an der Repräsentativität wurden im Artikel unter anderem als Selektionseffekte erläutert und begründet, warum diese in der Analyse der Daten vernachlässigt werden können (Stichwort: Prävalenzrange). Außerdem wurde in den Limitationen darauf hingewiesen, dass eine einfache Gewichtung der Daten vorgenommen wurde. Totalerhebungen, wie sie in England durch den National Health Service (NHS) durchgeführt werden, sind in Deutschland aufgrund des Fehlens einer vergleichbaren Datenbank nicht möglich.

## Zu: Repräsentativität der Stichprobe

Auch die Anmerkung der Verzerrungen aufgrund von geflüchteten Kindern, bei denen häufig der 01.01. des Geburtsjahres als Geburtstag angegeben wird, entspricht explizit der Darstellung im Originalartikel.

Zum Kommentar zu den onkologischen Diagnosen und der fehlenden Darstellung der Konfidenzintervalle ist anzumerken, dass dies der Entscheidung geschuldet ist, die Ergebnisse komprimiert darzustellen. Tab. 1 enthält eine Übersicht zu den Ergebnissen der onkologischen Daten mit den entsprechenden Konfidenzintervallen.

**Table 1:** 

Jahr	Patienten InGef	Versicherte InGef	Pro 100.000	95 % KI Untergrenze	95 % KI Obergrenze
2014	3651	1.167.826	313	303	323
2015	3597	1.175.289	306	296	316
2016	3535	1.159.625	305	295	315
2017	3573	1.175.692	304	294	314
2018	3559	1.184.627	300	291	310
2019	3516	1.168.257	301	291	311

*InGef* Institut für angewandte Gesundheitsforschung

In den Limitationen der Studie werden Einschränkungen im Hinblick auf nicht umsetzbare externe Validitätsprüfungen beschrieben. Der in Anlehnung an Fraser et al. verwendeten und frei zugänglichen Diagnoseliste des Projektes PraeKids ist zu entnehmen, dass es bei den ausgewählten Kodierungen, die der Prävalenzberechnung zugrunde liegen, um kein Abbild des Kindeskrebsregisters (KKR) handelt. So werden zwar beispielsweise die relevanten Codes C00-97 *Bösartige Neubildungen* erfasst, die im KKR gelistet werden. Die der PraeKids-Studie zugrunde liegende Code-Liste enthält jedoch auch den Code D48 – *Neubildung unsicheren oder unbekannten Verhaltens an sonstigen und nicht näher bezeichneten Lokalisationen* (38.403 Betroffene – GKV-SV). Dieser Code zählt beispielsweise in anderen Ländern wie der Schweiz durchaus zu den meldepflichtigen Diagnosen [8], wird aber nicht in den Meldelisten der deutschen Bundesländer aufgeführt (exemplarisch: NRW oder Rheinland-Pfalz [9, 10]). Über die Zuordnung einzelner Diagnosen zu den meldepflichtigen onkologischen Erkrankungen bestehen somit unterschiedliche fachliche Einschätzungen. Eine international konsentierte Code-Liste existiert nicht. Aufgrund des erwartbaren Abgleichs der Daten mit denen des KKR verweisen die Autor:innen im Artikel darauf, dass die beiden Datensätze voneinander abweichen. Ein direkter Vergleich ist aus dargestellten Gründen jedoch nicht möglich. Die Repräsentativität der Gesamtstudie aufgrund der onkologischen Diagnosen infrage zu stellen, halten die Autor:innen entsprechend für nicht angemessen. Im Kommentar wird angeführt, dass aktuell höhere Überlebensraten von Krebspatient:innen zu berücksichtigen sind. Hierzu sei auf die Aussage der Autor:innen verwiesen, dass bei bestimmten Diagnosen ein Krankheitsverlauf möglich ist, der von einem akuten Zustand in einen chronischen Restzustand übergeht. Die Entscheidung, Kinder und Jugendliche mit diesen chronischen Restzuständen zu berücksichtigen, wurde in Absprache mit den beteiligten Palliativmediziner:innen im Rahmen des Studienverlaufs getroffen.

Zum Kommentar bzgl. der Berücksichtigung von „Chronikerpauschalen“ sei angemerkt, dass im Originalbeitrag der Autor:innen darauf hingewiesen wird, dass bei Sekundärdatenanalysen anhand von ICD-Codes die Gefahr des „Upcodings hinsichtlich der Abrechnungsrelevanz und leistungsorientierten Vergütung eine Rolle“ spielt. Sollten aus diesem Grund Abrechnungsdaten des GKV-SV nicht mehr in Studien Verwendung finden dürfen, hätte dies gravierende Auswirkungen auf die Versorgungsforschung. Vielmehr ist es gute wissenschaftliche Praxis auf diesen möglichen Umstand hinzuweisen – wie sowohl im Studienbericht als auch im Originalbeitrag geschehen.

## Zu: Filterung der Daten mit verschiedenen Listen von ICD-10-Codes

Die Auswahl der Kodierungen hat, wie beschrieben, in der Zusammenarbeit mit pädiatrischen Palliativmediziner:innen und mittels verschiedener Rückkopplungsschleifen stattgefunden. Im Artikel verweisen die Autor:innen auf die Downloadmöglichkeit dieser Liste.

Adaption und Erweiterung der Fraser-Liste wurden zudem im Fachartikel begründet (Aktualisierung der ICD-10; Ausschluss nicht lebensbedrohlicher bzw. lebensverkürzender Diagnosen). In der Gruppendiskussion der Palliativmediziner:innen wurden anhand deren fachlicher Einschätzungen entsprechende Diagnosen für die Erhebung eliminiert oder hinzugefügt. Die jeweiligen Ein- bzw. Ausschlusskriterien finden sich im Artikel.

Möglich wäre gewesen, die einzelnen ergänzten Häufigkeiten zu nennen und jeweils kritisch zu diskutieren. Ziel dieser Studie war jedoch die Erfassung der Gesamt-Prävalenzzahl, weshalb ein Forschungsdesign entwickelt wurde, dass die zentralen Einschlusskriterien Lebensbedrohung, Lebensverkürzung und Eingruppierung in TfSL-1‑4 beinhaltete. Es sei zu diesem Hinweis aber auch angemerkt, dass die von Fraser et al. in ihren unterschiedlichen Studien verwendeten ICD-10-Codes weder bei dem Transfer auf die bislang verwendete Prävalenz von 50.000, noch bei der im Kommentar benannten Anzahl von 100.000 geprüft, hinterfragt bzw. modifiziert wurden. Warum dieser inhaltlich durchaus nachvollziehbare Anspruch ausschließlich an die modifizierten Code-Listen der PraeKids-Studie gestellt wird, bleibt im Kommentar unaufgelöst.

Ihr Hinweis auf die einzelnen 4‑stelligen Kodierungen bezüglich des Codes E74 *Sonstige Störungen des Kohlenhydratstoffwechsels *ist korrekt, hätte bei Anwendung jedoch für alle Diagnosen gleichermaßen erfolgen müssen, was forschungsökonomisch nicht umsetzbar gewesen wäre. Auch auf diese Limitierung bei der Nutzung der ICD-10 als klassifikatorisches System ist durch die PraeKids-Autor:innen sehr deutlich hingewiesen worden.

Zu den Anmerkungen bezüglich der Corona-Diagnosen ist festzuhalten, dass die Studie Daten bis 2019 erhoben hat. Zu diesem Zeitpunkt der Kodierungen und der Auswertung konnte keine gesicherte Aussage über den Verlauf einer Corona-Infektion im Kindes- und Jugendalter getroffen werden. Auch dieser Code wurde explizit mit den Palliativmediziner:innen in der Gruppendiskussion besprochen und aufgrund deren fachlicher Einschätzung bei der Erhebung berücksichtigt. Im Jahr 2019 wurden hierzu insgesamt 311 Diagnosen (GKV-SV) gestellt, die in die Prävalenzberechnung eingeflossen sind. Mit Blick auf die Gesamtprävalenz ist diese Fallzahl zu vernachlässigen. Zudem weisen die Autor:innen darauf hin, dass es bei diesen Entscheidungen ebenso wie bei sämtlichen Diagnosestellungen immer Varianzen in den Krankheitsverläufen sowie sich verändernde Therapiemöglichkeiten zu berücksichtigen gilt. Für Folgestudien wäre dieser Code auf Grundlage der nun verfügbaren Studienlage erneut kritisch zu prüfen bzw. aus der Liste zu entfernen.

Die schraffierten Flächen in Abb. 4 bei TfSL1 und TfSL4 wurden damit begründet, dass lebensbedrohliche Erkrankungen erfasst wurden, die nicht zwingend zu einem frühen Tod und einer palliativen Versorgung führen müssen. Um genaue zahlenmäßige Aussagen treffen zu können, wie im Kommentar gewünscht, hätten Mortalitätszahlen aller Erkrankungen vorliegen bzw. erhoben werden müssen. Ziel der Studie war jedoch die Erhebung der Prävalenz lebensverkürzender und lebensbedrohlicher Erkrankungen. Anhand der Schraffierungen haben die Autor:innen schaubildlich verdeutlicht, dass die Daten Diagnosen enthalten, die zwar lebensbedrohlich, aber bei günstigem Therapieverlauf heilbar sind. Ihr Hinweis auf die Überlebensrate von 94,7 % bei der akuten lymphatischen Leukämie weist im Zusammenspiel mit anderen, weniger erfolgreich therapierbaren Erkrankungen aus TfSL1 auf eine Tendenz, die die Schraffierung in diesem Feld visuell andeutet. Weniger exakt sind hier Prognosen zu Krankheitsverläufen zu Diagnosen aus TfSL4 möglich, da hier auch viele nicht spezifischer diagnostizierte neurologische Erkrankungen im Sinner komplexer Behinderungen subsumiert sind, die ein frühes Versterben bedingen können, bei denen aber auch steigende Lebenserwartungen zu verzeichnen sind [11].

Die Autorin und der Autor der PraeKids-Studie teilen das Interesse der Verfasser:innen des Kommentars an vertiefenden Studien, die auf der Grundlage der nun vorliegenden Diagnoseprävalenzen Versorgungsbedarfe in unterschiedlichen Krankheitsphasen erfassen und analysieren. Den an PraeKids beteiligten engagierten Expert:innen aus der pädiatrischen Palliativversorgung und der Versorgungsforschung sei für ihre Kooperation ausdrücklich gedankt.

## Footnotes

1 TfSL: Together-for-Short-Lives, Diagnosegruppierung.

## References

[1] Bundesverband Kinderhospiz (2022) Über uns. https://bundesverband-kinderhospiz.de/ueberuns. Zugegriffen: 2. Mai 2022

[2] Jennessen S, Hurth S (2021). Der Qualitätsindex für Kinder- und Jugendhospizarbeit.

[3] Zernikow B, Gertz B, Hasan C (2017). Pädiatrische Palliativversorgung – herausfordernd anders. Aufgaben, Ziele und Besonderheiten. Bundesgesundheitsblatt Gesundheitsforschung Gesundheitsschutz.

[4] Fraser LK, Miller M, Hain R, Norman P, Aldridge J, McKinney PA, Parslow RC (2012). Rising national prevalence of life-limiting conditions in children in England. Pediatrics.

[5] Zylbersztejn A, Gilbert R, Hjern A, Wijlaars L, Hardelid P (2018). Child mortality in England compared with Sweden: a&nbsp;birth cohort study. Lancet.

[6] Wang H, Liddell CA, Coates MM (2014). Global, regional, and national levels of neonatal, infant, and under-5 mortality during 1990–2013: a systematic analysis for the Global Burden of Disease Study 2013. Lancet.

[7] Fraser LK, Gibson-Smith D, Jarvis S, Norman P, Parslow RC (2020). Estimating the current and future prevalence of life-limiting conditions in children in England. Palliat Med.

[8] Kinderkrebsregister Schweiz Zu meldende Krebserkrankungen. https://www.kinderkrebsregister.ch/meldepflichtige/was-muss-gemeldet-werden/

[9] Landeskrebsregister NRW (2016) Meldepflichtige Diagnosen an das Landeskrebsregister NRW. https://www.landeskrebsregister.nrw/fileadmin/user_upload/dokumente/melderinformation/MeldepflichtigeDiagnosen_LKR-NRW_10.06.2021.pdf

[10] Krebsregister Rheinland-Pfalz (2022) Welche Diagnosen sind meldepflichtig? https://www.krebsregister-rlp.de/fuer-melder/faq-haeufig-gestellte-fragen/1-allgemeine-fragen-und-meldepflicht/welche-diagnosen-sind-meldepflichtig

[11] Pitsch HJ, Thümmel I (2017). Lebenschancen für Menschen mit geistiger Behinderung im Alter.

